# Inflammatory Pathways and Immune Microenvironment in Non‐Small Cell Lung Cancer: Multi‐Dimensional Analysis and Machine Learning Prediction

**DOI:** 10.1111/jcmm.70689

**Published:** 2025-07-19

**Authors:** Yuan Fang, Yuli Wang, Lanlan Yang, Si Yuan, Jiefei Gu, Ziyi Zhou, Zhihong Fang, Yan Li

**Affiliations:** ^1^ Clinical Medical Center of Oncology, Shanghai Municipal Hospital of Traditional Chinese Medicine Shanghai University of Traditional Chinese Medicine Shanghai China; ^2^ Department of Oncology Nujiangzhou Hospital of Traditional Chinese Medicine Yunnan China; ^3^ Information Center, Shanghai Municipal Hospital of Traditional Chinese Medicine Shanghai University of Traditional Chinese Medicine Shanghai China

**Keywords:** biomarkers, cell cycle, immune microenvironment, inflammatory factors, machine learning, Mendelian randomisation, non‐small cell lung cancer

## Abstract

Non‐small cell lung cancer (NSCLC) is a highly complex malignancy involving multiple molecular pathways including inflammatory responses, immune regulation and cell cycle dysregulation. Although previous studies have indicated the important role of inflammatory factors in NSCLC pathogenesis, the causal relationship between specific inflammatory factors and NSCLC risk, as well as their interactions with the immune microenvironment, has not been comprehensively elucidated. This study systematically evaluated the causal relationship between various inflammatory factors and NSCLC risk using Mendelian randomisation (MR) methodology. Through comprehensive transcriptomic analysis, network pharmacology approaches and protein–protein interaction network construction, we revealed molecular targets and key pathways in NSCLC. Additionally, we applied machine learning models to predict NSCLC and analysed the correlation between immune cell composition and cell cycle regulatory genes in NSCLC using flow cytometry. MR analysis showed that TGFB1 and CCL11 were positively correlated with NSCLC risk (OR = 1.173, *p* = 0.020; OR = 1.192, *p* = 0.003), while CD40 and CCL4 demonstrated protective effects (OR = 0.857, *p* = 0.015; OR = 0.896, *p* = 0.049). Bioinformatic analysis identified 74 overlapping drug‐disease targets enriched in multiple inflammation‐related signalling pathways. Machine learning models performed well in predicting NSCLC with AUC values of 0.723–0.763. Immune cell analysis revealed significantly increased CD8+ *T* cells and regulatory *T* cells (Tregs) in NSCLC samples, while naïve *B* cells were decreased. Complex correlations existed between cell cycle regulatory genes and immune cell composition, with CDK2 and CDK3 negatively correlated with Tregs (*R* = −0.8, *p* = 0.014; *R* = −0.72, *p* = 0.037), while CDK5 positively correlated with Tregs (*R* = 0.8, *p* = 0.014). This study revealed genetic associations between specific inflammatory factors and NSCLC risk, elucidating the complex interactions between inflammatory pathways and the immune microenvironment in NSCLC pathogenesis.

## 1 Introduction

Non‐small cell lung cancer (NSCLC) constitutes approximately 85% of lung malignancies globally, maintaining devastating mortality rates that underscore the urgent need for deeper mechanistic understanding. The multifaceted aetiology of NSCLC encompasses intricate molecular networks, where inflammatory processes have emerged as fundamental orchestrators of tumorigenesis, disease progression and metastatic dissemination [1, 2, 3].

Non‐small cell lung cancer (NSCLC) constitutes approximately 85% of lung malignancies globally, maintaining devastating mortality rates that underscore the urgent need for deeper mechanistic understanding. The multifaceted aetiology of NSCLC encompasses intricate molecular networks, where inflammatory processes have emerged as fundamental orchestrators of tumorigenesis, disease progression and metastatic dissemination [4, 5, 6]. Conventional epidemiological investigations have encountered substantial limitations due to confounding variables and bidirectional causality concerns, hampering efforts to establish unequivocal causal links between inflammatory biomarkers and malignant transformation.

Mendelian randomisation (MR) methodology has revolutionised genetic epidemiology by employing genetic polymorphisms as instrumental variables to infer causal associations between risk factors and disease outcomes, thereby circumventing traditional confounding biases. This innovative approach offers unprecedented opportunities to dissect the causal contributions of inflammatory mediators to NSCLC pathogenesis [7, 8, 9].

The NSCLC tumour microenvironment (TME) represents a dynamic ecosystem shaped by intricate cross‐talk between neoplastic cells and diverse immune effector populations, encompassing T‐cell subsets, B‐lymphocytes, myeloid‐derived cells and innate immune components. These immune cellular networks critically determine disease trajectory and therapeutic susceptibility through their functional polarisation and spatial organisation [10, 11, 12]. While emerging investigations have documented profound immune dysregulation in NSCLC, the comprehensive characterisation of immune architectural patterns and their mechanistic integration with inflammatory cascades demands further investigation.

Aberrant cell cycle control constitutes a cancer hallmark, with cyclin‐dependent kinases (CDKs) serving as master regulators of proliferative homeostasis. The emerging recognition of bidirectional communication between cell cycle machinery and immune surveillance mechanisms within the TME represents a paradigm shift with transformative therapeutic implications [13, 14, 15]. Understanding how these interconnected networks—inflammation, immune regulation and cell cycle control—collectively shape NSCLC biology may unlock novel precision medicine strategies.

This study aims to comprehensively investigate the inflammatory landscape of NSCLC through an integrated approach combining Mendelian randomisation, transcriptomic analysis, network pharmacology and immune cell profiling. By establishing causal relationships between specific inflammatory factors and NSCLC risk, identifying key molecular targets and pathways, and characterising the immune microenvironment in relation to cell cycle regulation, we seek to provide novel insights into NSCLC pathogenesis and uncover potential therapeutic targets for this devastating disease.

## 2 Methods

### 2.1 Study Design and Data Sources

This investigation utilised an integrative multi‐layered analytical framework to explore inflammatory networks and immune landscape dynamics in non‐small cell lung cancer (NSCLC). The research strategy combined genetic epidemiological approaches, transcriptomic profiling, computational biology, systems pharmacology and artificial intelligence techniques to achieve comprehensive mechanistic insights into NSCLC development. Genomic information was extracted from diverse public repositories, encompassing genome‐wide association study (GWAS) databases, Gene Expression Omnibus (GEO) platforms and The Cancer Genome Atlas (TCGA) collections covering lung adenocarcinoma and squamous cell carcinoma cohorts [16, 17].

### 2.2 Mendelian Randomisation Analysis

Causal relationships between inflammatory mediators and NSCLC susceptibility were investigated through Mendelian randomisation (MR) methodology. Genetic variants (single nucleotide polymorphisms, SNPs) linked to circulating concentrations of 13 inflammatory markers (encompassing CDKL1, CXCL8, TGFB1, CD40, CCL2, CCL4, CCL7, CCL8, CCL11, CCL13, among others) served as instrumental variables. SNP selection criteria required genome‐wide statistical significance (*p* < 5 × 10^−8^) from extensive GWAS repositories [18, 19]. Instrumental variable validation encompassed verification of the three fundamental MR principles: Robust exposure‐instrument association, absence of confounder‐instrument relationships and exclusive outcome influence via exposure pathways. Linkage disequilibrium (LD) clumping eliminated correlated variants (*r* ^2^ < 0.001) to maintain instrument independence.

### 2.3 Statistical Methods for Causal Inference

Robust causal estimation employed multiple complementary MR techniques: Inverse Variance Weighted (IVW) methodology served as the principal analytical approach, MR‐Egger regression detected horizontal pleiotropic effects, weighted median estimation maintained validity despite up to 50% invalid instruments and simple mode analysis provided additional sensitivity assessment. Causal effect magnitudes were expressed as odds ratios (ORs) with corresponding 95% confidence intervals (CIs) for each inflammatory factor's NSCLC risk association. Between‐instrument heterogeneity was quantified using Cochran's Q statistic, while pleiotropy detection utilised MR‐Egger intercept evaluation. Comprehensive visualisation included forest plots, scatter diagrams and funnel plots to illustrate findings and identify potential analytical biases.

### 2.4 Transcriptomic Data Processing and Analysis

Paired NSCLC tumour‐normal tissue RNA‐sequencing datasets were acquired from established public databases. Data preprocessing involved comprehensive quality assessment, adapter sequence removal and low‐quality read filtering. Processed sequences underwent alignment to the human reference genome (GRCh38) via HISAT2, followed by gene expression quantification through StringTie algorithms. Technical batch variation was corrected using ComBat normalisation procedures. Differential expression profiling between malignant and control specimens utilised DESeq2 with stringent criteria: |log2 fold change| > 1 and false discovery rate‐adjusted *p* < 0.05. Sample relationship patterns were explored through principal component analysis (PCA), while expression data distribution was visualised via volcano plots and hierarchical clustering heatmaps.

### 2.5 Network Pharmacology and Molecular Docking

NSCLC‐relevant molecular targets were systematically identified through Traditional Chinese Medicine Systems Pharmacology (TCMSP) and Genecord database mining. Drug–target and disease–target intersection analysis employed Venn diagram approaches to identify overlapping therapeutic opportunities. Protein–protein interaction (PPI) network construction utilised STRING database resources with confidence thresholds ≥ 0.7. Network topology analysis and visualisation were accomplished using Cytoscape software platforms. Selected therapeutic compounds (*n* = 6) underwent computational molecular docking evaluation to assess target protein binding characteristics using AutoDock Vina algorithms. Binding interaction analysis encompassed energy calculations and interaction pattern characterisation, including polar, electrostatic, hydrophobic and specific receptor–ligand contact identification.

### 2.6 Pathway Enrichment and Functional Analysis

Biological pathway significance was evaluated through Gene Ontology (GO) and Kyoto Encyclopedia of Genes and Genomes (KEGG) enrichment analyses using clusterProfiler R framework. Statistically significant biological processes, molecular functions, cellular localisations and signalling pathways were identified using adjusted *p* value thresholds < 0.05. Enrichment visualisationvisualization utilised dot plot representations, with colour gradients indicating statistical significance and point dimensions reflecting gene participation counts.

### 2.7 Machine Learning Model Development and Evaluation

NSCLC classification prediction models were developed using diverse machine learning architectures: Random forest (RF), support vector machine (SVM), gradient boosting machine (GBM), Lasso regression and hybrid ensemble methodologies based on transcriptomic signatures. Data partitioning allocated 80% for model training and 20% for internal validation. Feature optimisation employed recursive feature elimination with cross‐validation strategies. Model hyperparameter tuning utilised grid search optimisation with 10‐fold cross‐validation protocols. Performance assessment included accuracy, sensitivity, specificity, area under the receiver operating characteristic curve (AUC), confusion matrix analysis and 95% confidence interval calculations. External validation was conducted on independent datasets (GSE278343) to evaluate model generalisability.

### 2.8 Immune Cell Composition and Microenvironment Analysis

Immune cell subset proportions (22 distinct populations) within NSCLC and control tissues were computationally estimated using CIBERSORT deconvolution algorithms applied to transcriptomic data. Analysed populations encompassed B‐cell subtypes, T‐lymphocyte variants, natural killer cells, macrophage polarisation states, dendritic cell subsets and mast cell populations. Algorithm execution included 1000 permutation cycles for statistical significance determination. Inter‐group immune composition differences were assessed using non‐parametric Mann–Whitney *U* testing with Benjamini–Hochberg multiple comparison adjustment. Immune profile visualisation employed stacked bar charts displaying relative cellular percentages. Inter‐population correlation patterns were analysed using Spearman's rank correlation with hierarchical clustering heatmap representation.

### 2.9 Cell Cycle Gene and Immune Cell Correlation Analysis

Associations between cyclin‐dependent kinase (CDK) gene expression patterns (CDK2, CDK3, CDK4, CDK5, CDK6) plus CD209 and immune cellular populations were quantified using Pearson correlation coefficients. A statistical significance threshold was established at *p* < 0.05. Correlation visualisation incorporated scatter plot arrays and network diagrams employing red‐blue colour schemes (positive–negative correlations) with connection thickness proportional to correlation magnitude. Biological interpretation of observed associations considered established literature regarding cell cycle control mechanisms and immune cell functionality in malignancy, with particular emphasis on CDK‐regulatory *T* cell, CDK‐naive *B* cell, and other significant immune subset relationships.

### 2.10 Statistical Analysis and Data Visualisation

Comprehensive statistical analyses were executed using R statistical environment version 4.1.0. Continuous variable comparisons employed Student's *t*‐tests or appropriate non‐parametric alternatives. Multiple hypothesis testing corrections utilised Benjamini–Hochberg false discovery rate control procedures. Statistical significance criteria were maintained at adjusted *p* < 0.05. Data visualisation was accomplished using ggplot2, pheatmap, and ComplexHeatmap R packages for publication‐quality graphics generation.

## 3 Results

### 3.1 Causal Association Between Inflammatory Factors and Non‐Small Cell Lung Cancer Risk

**FIGURE 1:**
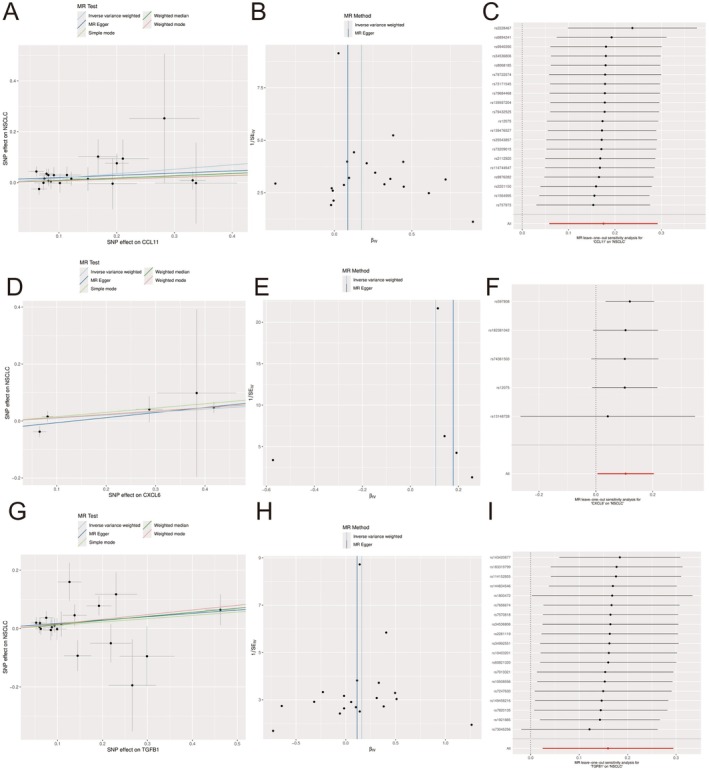
Causal association between inflammatory factors and non‐small cell lung cancer risk. (A–I) This study examined whether three inflammatory factors (CDKL1, CXCL8 and TGFB1) directly cause non‐small cell lung cancer using genetic data. The positive trends seen in the scatter plots suggest these inflammatory markers may indeed increase lung cancer risk. The analysis used multiple statistical methods to ensure reliability, with consistent results across different genetic variants. This genetic evidence provides new insights into how inflammation might contribute to lung cancer development and suggests potential new targets for prevention and treatment strategies.

This study systematically evaluated the potential causal relationship between three key inflammatory factors (CDKL1, CXCL8 and TGFB1) and the risk of non‐small cell lung cancer (NSCLC) using Mendelian randomisation (MR) methodology. The analysis results shown in the figure contain three sets of data, each targeting a specific inflammatory marker. The scatter plots (Figure 1A,D,G) show the relationship between the effects of various single nucleotide polymorphisms (SNPs) on inflammatory factor expression levels and NSCLC risk, with multiple regression lines representing different statistical methods, including inverse variance weighted method, MR‐Egger regression, weighted median method, and simple mode approach. The funnel plots (Figure 1B,E,H) assess the relationship between the precision of genetic instruments and the causal estimates of individual SNPs, helping to identify potential horizontal pleiotropy. The forest plots (Figure 1C,F,I) visually present the causal effects and confidence intervals of each independent SNP. The positive slope trends observed in most charts suggest that these inflammatory factors may play important roles in the pathogenesis of NSCLC, providing new genetic evidence for understanding the molecular mechanisms between inflammation and lung cancer development, while also opening potential directions for lung cancer prevention and treatment strategies targeting inflammatory pathways.

### 3.2 Analysis of the Association Between Inflammatory Factors and Non‐Small Cell Lung Cancer

**FIGURE 2:**
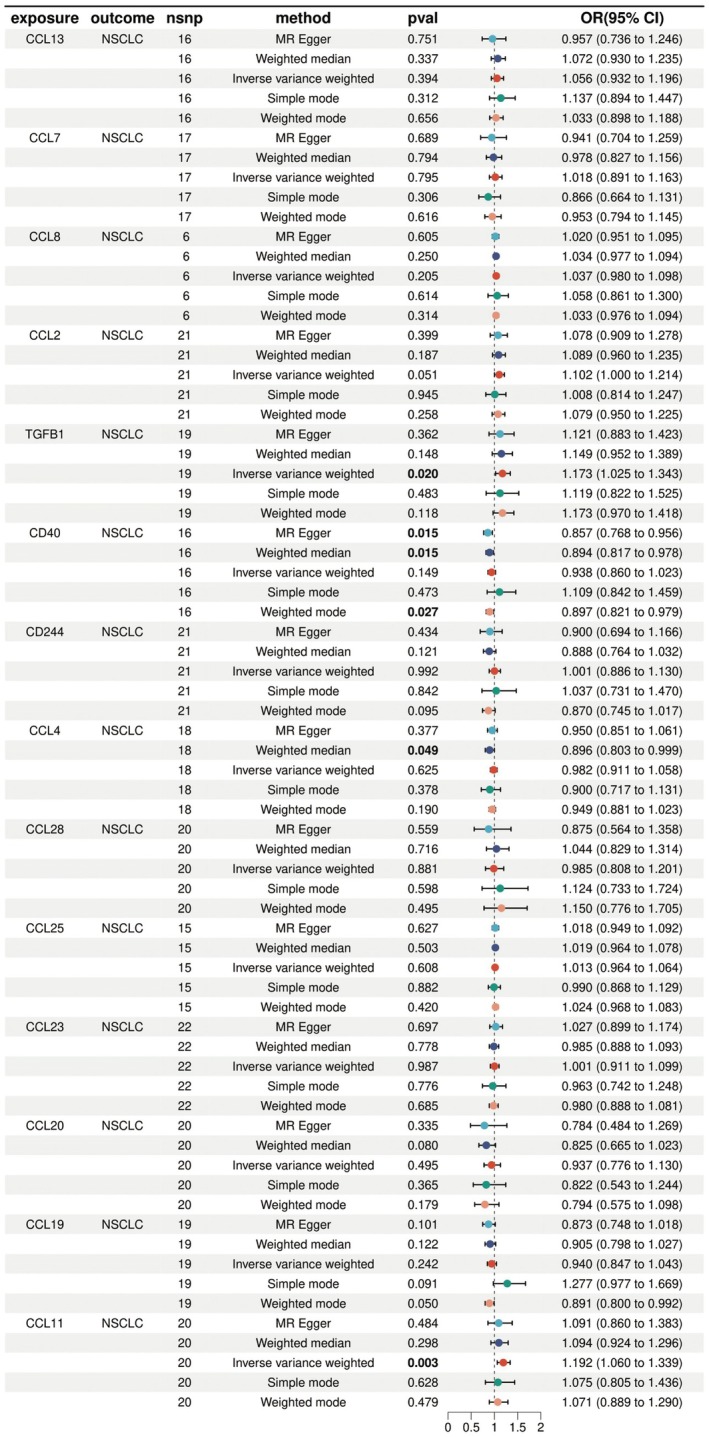
Analysis of the association between inflammatory factors and non‐small cell lung cancer. This study reveals that specific inflammatory factors have contrasting effects on lung cancer risk. TGFB1 and CCL11 increase non‐small cell lung cancer risk, with CCL11 showing the strongest harmful association. In contrast, CD40 and CCL4 appear protective against lung cancer. The remaining nine inflammatory factors tested showed no significant impact. These findings identify specific inflammatory molecules that could potentially serve as targets for lung cancer prevention or treatment strategies.

This study conducted a comprehensive analysis of the causal relationship between multiple chemokines and non‐small cell lung cancer (NSCLC) using Mendelian randomisation methods. The results showed that among the 13 inflammatory factors evaluated, 4 factors were statistically significantly associated with NSCLC risk. TGFB1 showed a positive correlation with NSCLC risk through inverse variance weighted analysis (OR = 1.173, 95% CI: 1.025–1.343, *p* = 0.020), suggesting that elevated TGFB1 levels may increase the risk of developing NSCLC. In contrast, CD40 demonstrated a protective effect through two analytical methods (MR Egger and weighted median), showing a negative correlation with NSCLC risk (OR = 0.857 and 0.894, both *p* = 0.015). CCL4 also showed a similar protective effect (OR = 0.896, *p* = 0.049). CCL11, similar to TGFB1, exhibited a promoting effect on NSCLC development (OR = 1.192, *p* = 0.003), representing the strongest statistical significance among all factors. CCL2's association was at the threshold of statistical significance (*p* = 0.051). No clear statistical associations were found between the remaining inflammatory factors such as CCL13, CCL7, CCL8 and so forth, and NSCLC risk. These findings not only reveal the differential roles of specific inflammatory factors in the pathogenesis of lung cancer but also provide valuable genetic evidence for lung cancer prevention and treatment strategies based on inflammatory pathways. Future research can further explore the molecular mechanisms of these key inflammatory factors to develop potential biomarkers or therapeutic targets (Figure 2).

### 3.3 Multi‐Dimensional Network Analysis of Molecular Targets and Pathways in Non‐Small Cell Lung Cancer

**FIGURE 3:**
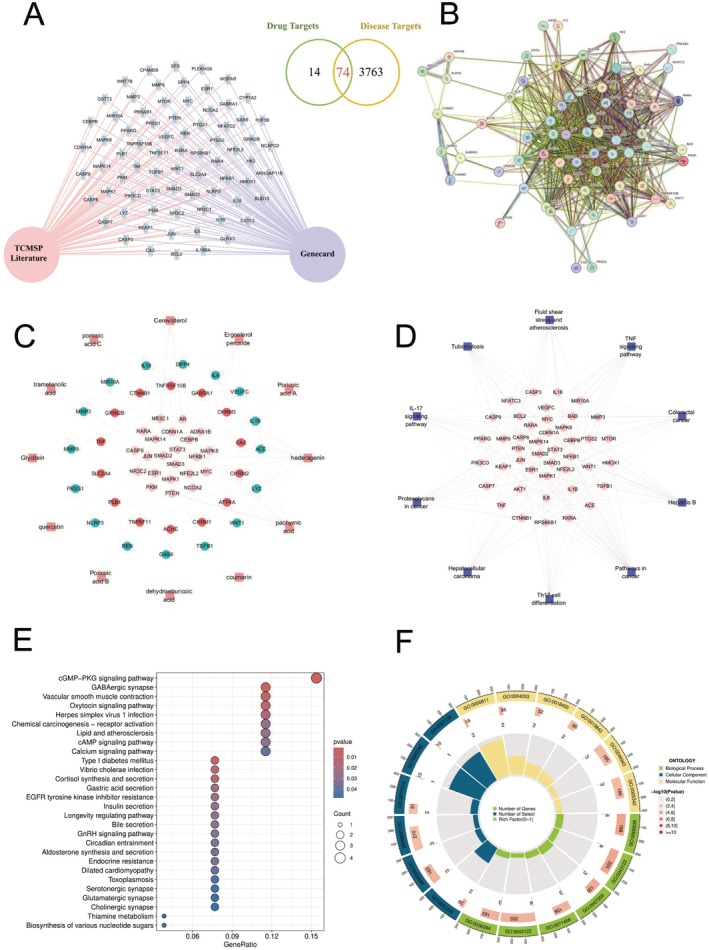
Multi‐dimensional network analysis of molecular targets and pathways in non‐small cell lung cancer. This figure shows how genes, proteins and pathways interact in non‐small cell lung cancer. (A) identifies 74 targets shared between drugs and the disease. (B) displays how these proteins connect with each other. (C) shows interactions between genes (red) and compounds (blue‐green). (D) maps key disease pathways, highlighting inflammation signals. (E) ranks the most significant biological pathways involved in lung cancer. (F) presents genomic locations linked to cancer functions. Together, these networks reveal potential treatment targets and emphasise how inflammation contributes to lung cancer development.

This figure presents a comprehensive multi‐dimensional analysis of molecular targets and networks in non‐small cell lung cancer (NSCLC). Figure 3A illustrates a bioinformatics network connecting drug targets derived from traditional Chinese medicine systems pharmacology (TCMSP) literature and the Genecord database, with a Venn diagram highlighting 74 overlapping targets between drug and disease associations, representing potential therapeutic intervention points. Figure 3B displays an intricate protein–protein interaction network where nodes of various colours represent different genes or proteins, with connecting edges indicating their functional relationships. Figure 3C shows a gene‐compound interaction network with red nodes representing gene targets and blue‐green nodes depicting compound molecules, surrounded by annotations of bioactive substances including polyglutamic acid and dehydroacetic acid. Figure 3D maps disease‐related pathways, demonstrating the involvement of multiple signalling cascades in NSCLC, including the IL‐17 pathway, TNF signalling, complement and coagulation cascades, along with inflammatory factors such as IL‐6, TNF and TGFB1. Figure 3E presents pathway enrichment analysis in dot plot format, with colour intensity and dot size indicating enrichment significance and gene count respectively; the most significantly enriched pathways include cGMP‐PKG signalling, GABAergic synapses and vascular smooth muscle contraction. Figure 3F features a circular diagram mapping genomic positions to functional annotations, with the outer ring showing chromosomal locations and inner rings representing different functional categories. This integrated analysis reveals the multi‐level molecular mechanisms of NSCLC, including key targets, signalling pathways, and drug action mechanisms, providing a systematic molecular foundation for developing new therapeutic strategies. The enrichment of multiple inflammation‐related signalling pathways particularly underscores the critical role of inflammatory processes in NSCLC development.

### 3.4 Non‐Small Cell Lung Cancer Targeted Drug Molecular Structures and Binding Site Analysis

**FIGURE 4:**
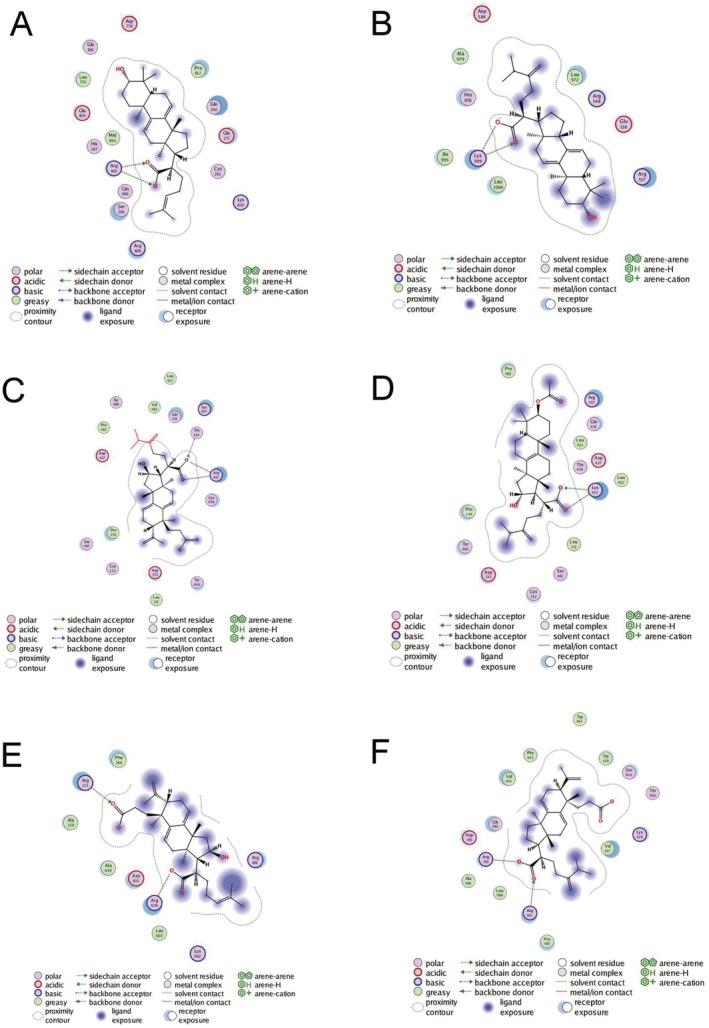
Non‐small cell lung cancer targeted drug molecular structures and binding site analysis. These images display six potential non‐small cell lung cancer targeted therapeutic compounds (A–F) and their molecular structures. Each structure uses a colour‐coding system to show specific binding patterns with protein targets, including polar, acidic, basic and hydrophobic interactions. Blue areas indicate exposure levels, while dotted contours mark molecular proximity regions. This detailed binding characteristic analysis helps researchers evaluate the potential effectiveness and specificity of these compounds as targeted drugs for non‐small cell lung cancer.

These images display six different molecular structures (Figure 4A–F), which are potential compounds for the treatment of non‐small cell lung cancer (NSCLC). Each panel shows a unique molecular structure and its interaction pattern with protein binding sites. These interactions are marked through a consistent colour‐coding system, including contacts with polar, acidic, basic and hydrophobic residues, as well as various receptor‐ligand interactions, such as sidechain acceptor/donor, backbone acceptor/donor, arene‐arene, arene‐H, and arene‐cation interactions. The blue gradient areas indicate the exposure degree of ligands and receptors, while the dotted contours represent the proximity regions around the molecules. These detailed interaction analyses are crucial for understanding how these compounds bind to targets related to non‐small cell lung cancer, thereby evaluating their effectiveness and specificity as potential therapeutic drugs.

### 3.5 Non‐Small Cell Lung Cancer Gene Expression Comparison Analysis Results

**FIGURE 5:**
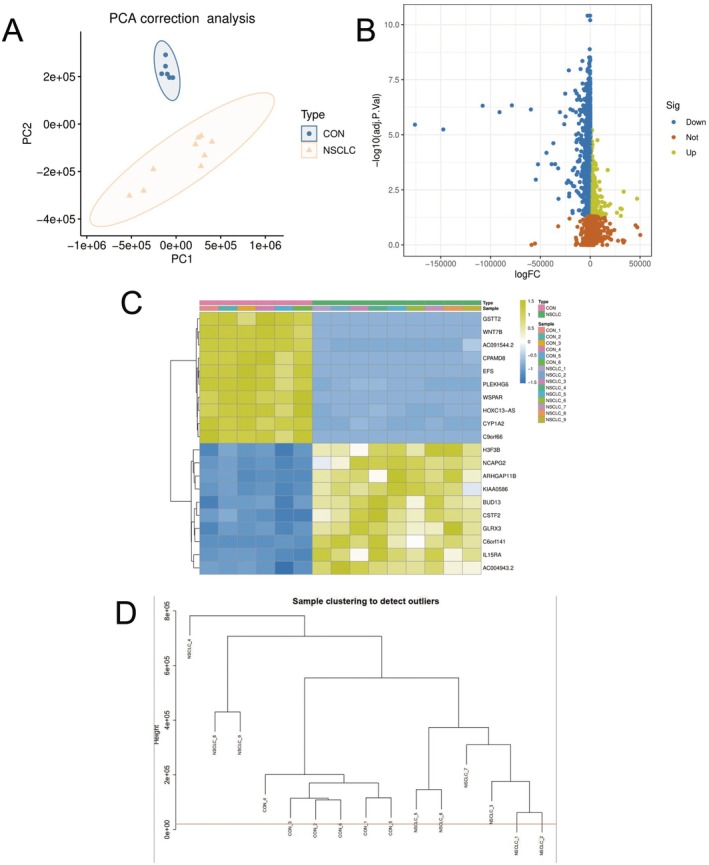
Non‐small cell lung cancer gene expression comparison analysis results. (A) PCA analysis displays two groups of samples forming distinctly separated clusters. (B) volcano plot exhibits numerous significantly differentially expressed genes, with blue indicating downregulation and yellow indicating upregulation. (C) heatmap reveals specific genes showing opposite expression patterns in cancer samples versus control group. (D) hierarchical clustering further confirms that samples naturally group according to disease status. Overall results indicate clear gene expression difference patterns between NSCLC and normal control groups.

Figure 5A shows PCA correction analysis: Principal component analysis results show clear differences in gene expression patterns between NSCLC samples (beige) and the control group (blue). The two sample groups form distinct clusters along PC1 and PC2 dimensions, indicating systematic gene expression changes due to disease state. Figure 5B shows volcano plot analysis: It shows the distribution of differentially expressed genes. Blue dots represent downregulated genes, yellow dots represent upregulated genes, and brown dots represent genes with no significant difference. The vertical axis (−log10 (*p* value)) reflects statistical significance, while the horizontal axis (logFC) shows the magnitude of expression change. The plot demonstrates numerous significantly differentially expressed genes between NSCLC and control groups. Figure 5C shows heatmap analysis: It displays expression levels of key differential genes across samples. Genes in the upper part of the heatmap show high expression (yellow) in NSCLC samples (green label) and low expression (blue) in the control group (blue label); genes in the lower part show the opposite expression pattern. This indicates the existence of gene expression signatures associated with NSCLC. Figure 5D shows sample clustering analysis: It detects outliers through hierarchical clustering, with the dendrogram showing similarity relationships between samples. NSCLC samples and control group samples generally cluster into different branches, further confirming the association between disease state and gene expression patterns. These results collectively reveal significant gene expression differences between NSCLC and normal control groups, providing a foundation for understanding the molecular mechanisms of NSCLC and identifying potential biomarkers.

### 3.6 Non‐Small Cell Lung Cancer Machine Learning Model Evaluation Results

**FIGURE 6:**
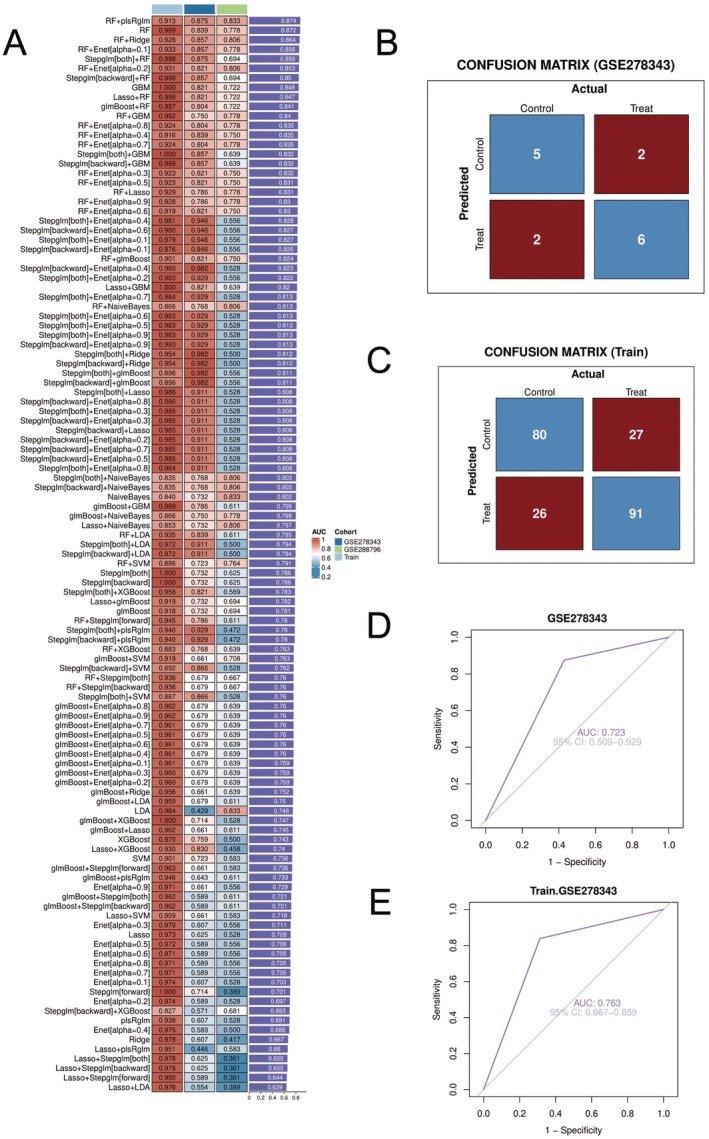
Non‐small cell lung cancer machine learning model evaluation results. The figure presents machine learning model performance for non‐small cell lung cancer classification. (A) shows a heatmap comparing various algorithms (RF, SVM, GBM) with AUC values mostly between 0.7–0.8. (B) and (C) display confusion matrices for test and training sets with accuracies of 73.3% and 76.3%, respectively. (D, E) show ROC curves with AUC values of 0.723 (test) and 0.763 (training). Overall, these models demonstrate good but imperfect predictive capability for non‐small cell lung cancer.

This figure shows the performance evaluation results of multiple machine learning classification models for non‐small cell lung cancer: Figure 6A displays a performance metric comparison of various machine learning algorithms and their combinations. The heatmap presents accuracy values for different algorithms (such as RF, SVM, GBM, Lasso, etc.) and their combinations, with colour intensity indicating performance levels. Based on the metrics, most models have AUC values between 0.7–0.8, indicating good classification capability for non‐small cell lung cancer. Figure 6B shows the confusion matrix on the GSE278343 dataset. The matrix indicates that the model correctly identified five samples in the control group and misidentified 2; in the treatment group, it correctly identified 6 samples and misidentified 2. The overall accuracy is (5+6)/(5+6+2+2) = 11/15≈73.3%. Figure 6C presents the confusion matrix performance on the training set. The model correctly predicted 80 cases in the control group and misclassified 27 as treatment group; in the treatment group, it correctly predicted 91 cases and misclassified 26 as control group. The training set accuracy is (80+91)/(80+91+27+26) = 171/224≈76.3%, showing good training effectiveness.

Figure 6D shows ROC curve analysis for the GSE278343 dataset, with an area under the curve (AUC) of 0.723 and a 95% confidence interval of 0.509–0.929. This indicates that the model has above‐average discrimination ability on the independent test set. Figure 6E: ROC curve analysis for the training set, with an AUC value of 0.763 and a 95% confidence interval of 0.687–0.832, indicating good discrimination ability on the training data. The overall results suggest that these machine learning models show promising potential in predictive classification of non‐small cell lung cancer, though there is room for improvement. The comparison of various algorithm combinations helps select the optimal model for clinical application.

### 3.7 Non‐Small Cell Lung Cancer Gene Expression and Related Pathway Analysis

**FIGURE 7:**
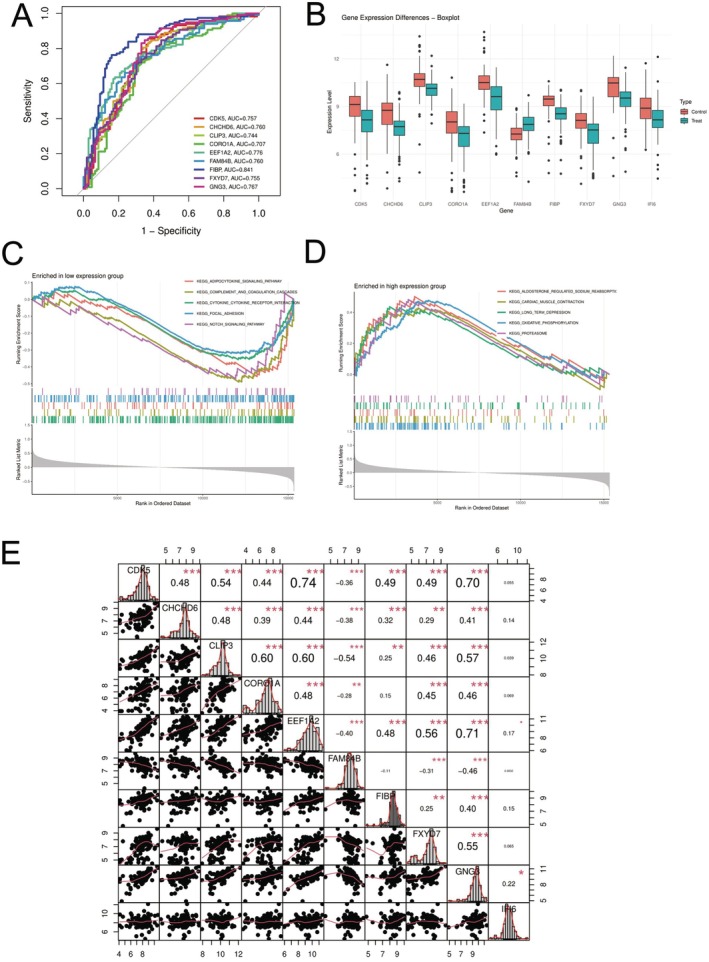
Non‐small cell lung cancer analysis summary. (A–E) This study found FIBP as the best diagnostic marker for non‐small cell lung cancer, with clear expression differences between tumour and normal samples. Different gene groups showed distinct pathway enrichments, and several genes demonstrated strong correlations. These findings identify potential biomarkers and therapeutic targets for improving non‐small cell lung cancer diagnosis and treatment.

Figure 7A shows ROC curve analysis of multiple genes in non‐small cell lung cancer diagnosis, with the FIBP gene (blue curve) showing the highest area under the curve, indicating its optimal diagnostic performance. Genes such as CDK5, CHCIHD6 and CLIP3 also demonstrate good diagnostic potential and could serve as potential biomarkers. Differential expression patterns: The boxplot in Figure 7B clearly illustrates gene expression differences between the control group (red) and tumour group (teal). Most studied genes show significant expression level changes between the two groups, providing key clues for understanding the molecular mechanisms of the disease. Pathway enrichment analysis: The low expression group (Figure 7C) shows enrichment in autophagosome signalling pathways, complement activation pathways, cytokine receptor interactions and focal adhesion pathways. In contrast, the high expression group (Figure 7D) is enriched in autophagosome regulation, oxidative phosphorylation and long‐term depression related pathways. These differences reflect complex molecular events in tumour tissues.

### 3.8 Immune Cell Profile Analysis in Non‐Small Cell Lung Cancer

**FIGURE 8:**
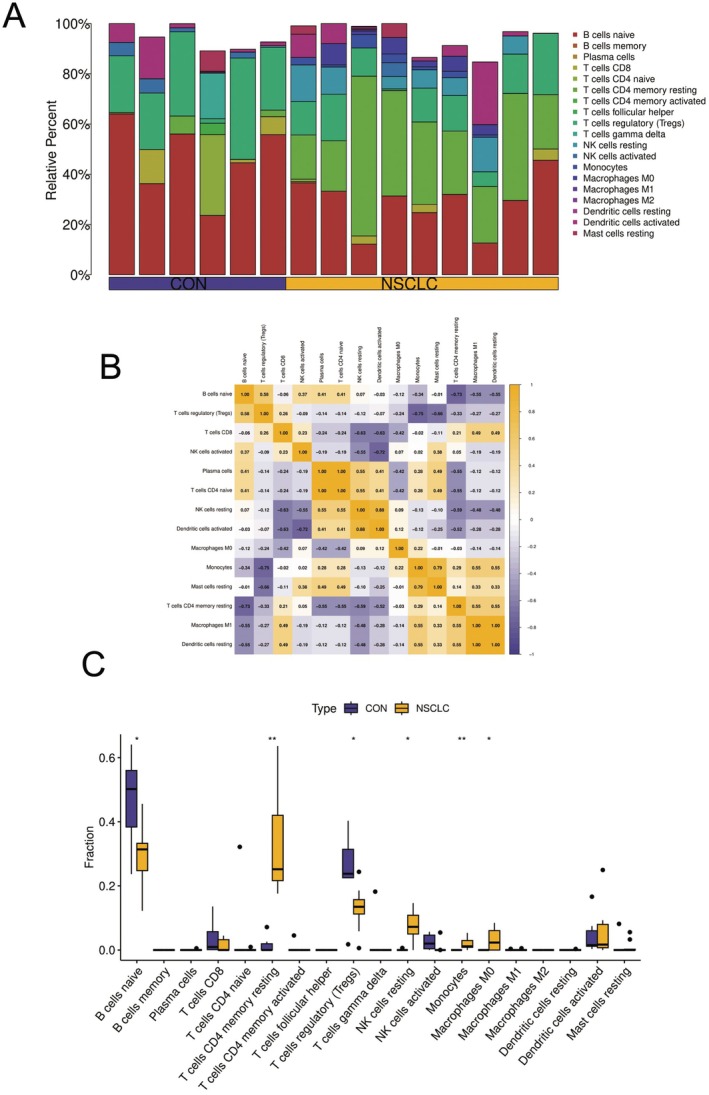
Immune cell profile analysis in non‐small cell lung cancer. A‐C In non‐small cell lung cancer, the immune environment shows fewer naive *B* cells but more CD8+ *T* cells, regulatory *T* cells and M0 macrophages. This pattern reveals competing forces at work: CD8+ *T* cells try to fight the tumour while regulatory *T* cells likely suppress this response. The relationships between these immune cells form a complex network that significantly differs from normal tissue, highlighting how immune system changes contribute to lung cancer development.

The figures present a comprehensive analysis of immune cell composition and interactions in non‐small cell lung cancer (NSCLC) compared to control samples (CON). Figure 8A displays stacked bar charts of relative immune cell percentages across samples, revealing significant differences between NSCLC and control groups. Notably, NSCLC samples show markedly decreased naive *B* cell proportions while exhibiting increased CD8+ *T* cells and regulatory *T* cells (Tregs). Additionally, macrophage subtypes (M0, M1 and M2) show altered distribution patterns, and dendritic cells appear more abundant in tumour samples. Figure 8B illustrates immune cell correlations through a heatmap ranging from negative (blue) to positive (yellow) associations. Key relationships include positive correlations between naive *B* cells and CD8+ *T* cells, strong positive associations between dendritic cells and M1 macrophages, negative correlations between various NK cell populations and CD8+ *T* cells, and notably strong positive correlations between resting and activated dendritic cells. Figure 8C provides statistical validation through boxplot comparisons between control (purple) and NSCLC (yellow) groups, confirming significant reductions in naive *B* cells and significant increases in CD8+ *T* cells, regulatory *T* cells and M0 macrophages in NSCLC samples. Both activated and resting NK cells also show significant differences between groups. These findings collectively demonstrate substantial remodelling of the tumour immune microenvironment in NSCLC, characterised by redistribution of *B* and *T* cell subsets and macrophage subtypes. The increased CD8+ *T* cells likely represent anti‐tumour immune responses, while elevated regulatory *T* cells may counteract this effect by suppressing immune activity and promoting tumour growth. These alterations reflect the complex interplay between tumour immune evasion mechanisms and host immune responses in the NSCLC microenvironment.

### 3.9 Correlations Between Cell Cycle Genes and Immune Cell Populations in the Tumour Microenvironment

The figures present significant correlations between cyclin‐dependent kinase (CDK) family gene expression and immune cell populations within the tumour microenvironment. CDK2 and CDK3 both demonstrate strong negative correlations with regulatory *T* cells (Tregs) (*R* = −0.8, *p* = 0.014 and *R* = −0.72, *p* = 0.037, respectively), indicating that as the expression of these genes increases, the proportion of immunosuppressive Tregs decreases. Similarly, both CDK3 and CDK4 show significant negative correlations with naive *B* cells (*R* = −0.77, *p* = 0.021 and *R* = −0.78, *p* = 0.017, respectively), suggesting coordinated influences of cell cycle regulators on multiple immune cell types.

Interestingly, CDK5 exhibits an opposite pattern with a strong positive correlation with Tregs (*R* = 0.8, *p* = 0.014), highlighting the complex and potentially contradictory roles different CDKs may play in immune regulation. Additionally, CD209, an important immune receptor, shows a significant positive correlation with resting mast cells (*R* = 0.78, *p* = 0.013), suggesting its potential involvement in tumour‐associated inflammatory responses.

**FIGURE 9:**
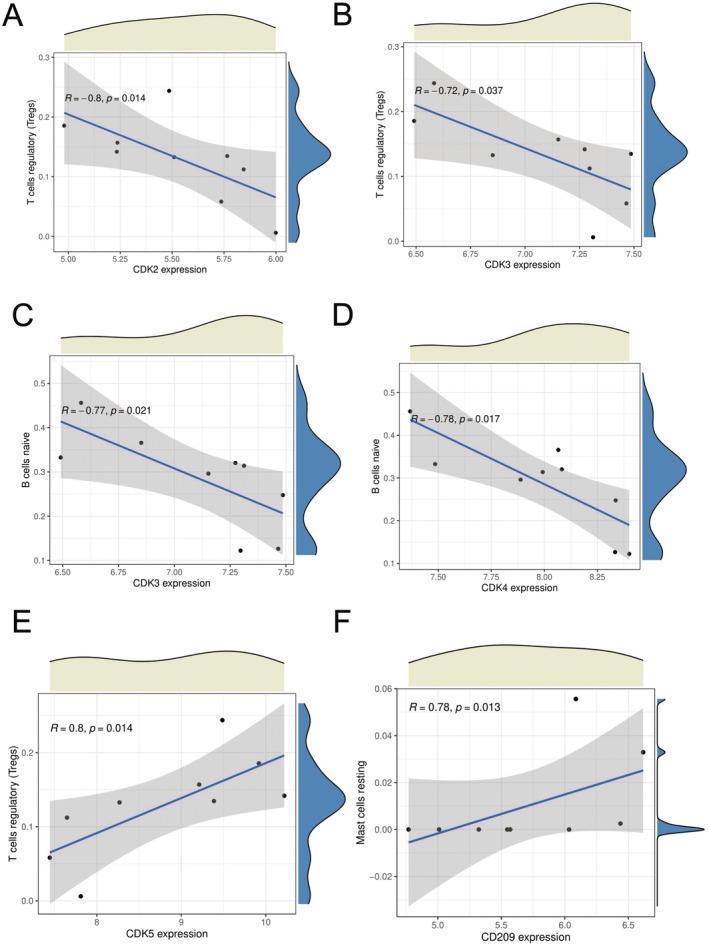
Correlations between cell cycle genes and immune cell populations in the tumour microenvironment. A‐F Cell cycle genes show strong relationships with immune cell populations in tumours. CDK2 and CDK3 negatively correlate with regulatory *T* cells, while CDK3 and CDK4 negatively correlate with naive *B* cells. In contrast, CDK5 positively correlates with regulatory *T* cells, and CD209 positively correlates with resting mast cells. These findings reveal that cell cycle regulators influence tumour immunity in complex ways, suggesting potential targets for cancer treatments that could affect both cell proliferation and immune responses.

These findings reveal important connections between cell cycle regulation and the tumour immune microenvironment. The differential relationships between various CDKs and immune cell populations suggest that cell cycle regulators may influence anti‐tumour immunity through multiple mechanisms. Understanding these correlations could provide valuable insights for developing therapeutic strategies targeting both cell cycle pathways and immune regulation in cancer treatment (Figure 9A–F).

### 3.10 Intricate Interplay Between Cell Cycle Regulators and Immune Cell Populations in Non‐Small Cell Lung Cancer

**FIGURE 10:**
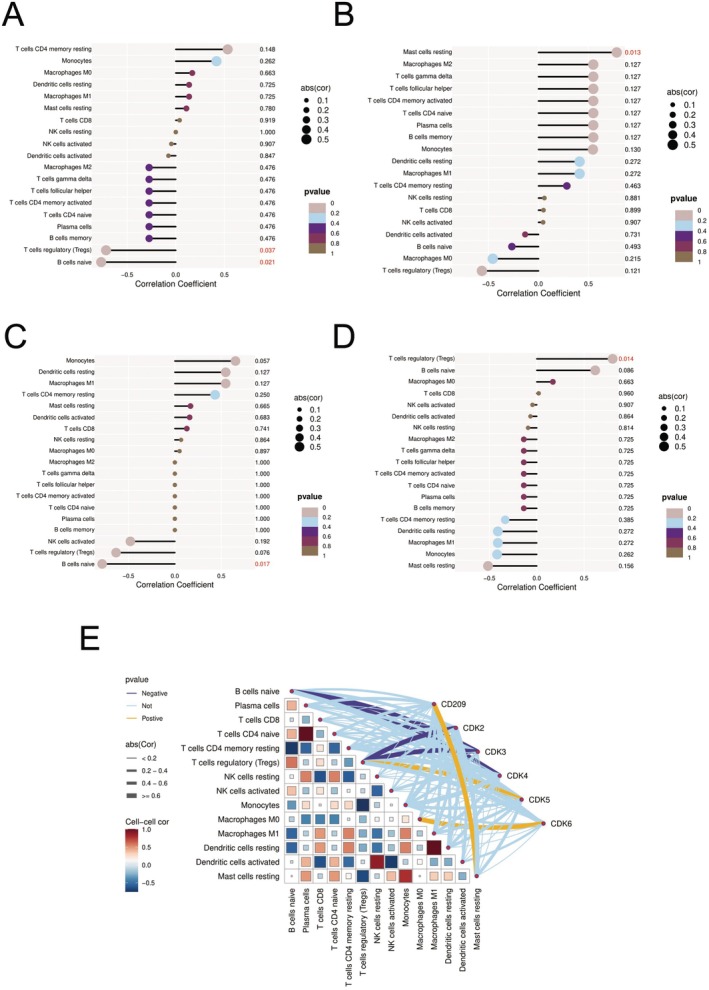
CDK genes and immune cells in lung cancer. (A–E) The images show how cell cycle genes (CDKs) relate to immune cells in lung cancer.CDK2: Negatively linked to regulatory *T* cells; positively linked to NK cells. CDK3: Positively linked to mast cells; negatively linked to dendritic cells. CDK4: Positively linked to various *T* cells; negatively linked to *B* cells. CDK5: Positively linked to both regulatory *T* cells and *B* cells. The network diagram shows these relationships visually, with different colours and line thicknesses showing correlation strength. Different CDKs affect immune cells in different ways, suggesting potential for treatments targeting both cell growth and immune response. RetryClaude can make mistakes. Please double‐check responses.

The figures present a comprehensive correlation analysis between cyclin‐dependent kinase (CDK) family genes and immune cell populations in non‐small cell lung cancer. Figure 10A reveals that CDK2 demonstrates a significant negative correlation with regulatory *T* cells (Tregs) (*p* = 0.027) while showing strong positive correlations with resting NK cells (correlation coefficient = 1.000), monocytes, M0 macrophages, and resting dendritic cells. Figure 10B indicates that CDK3 positively correlates with resting mast cells (correlation coefficient = 0.913), negatively correlates with activated dendritic cells (correlation coefficient approximately −0.731), and shows a moderate positive correlation with resting memory CD4+ *T* cells (correlation coefficient = 0.463). Figure 10C demonstrates that CDK4 positively correlates with monocytes (correlation coefficient = 0.657) and strongly correlates with multiple *T* cell subtypes including gamma delta *T* cells and follicular helper *T* cells (correlation coefficient = 1.000), while showing a significant negative correlation with naive *B* cells (*p* = 0.017). Figure 10D reveals that CDK5 exhibits a significant positive correlation with regulatory *T* cells (*p* = 0.014), a positive correlation with naive *B* cells (correlation coefficient = 0.386) and moderate positive correlations with various *T* cell subtypes.

Figure 10E provides a comprehensive network visualisation of these relationships, with colours ranging from red (positive correlation) to blue (negative correlation) and line thickness representing correlation strength. This complex network illustrates the multifaceted connections between CDK2‐6 and CD209 with various immune cell populations. These findings collectively reveal sophisticated relationships between cell cycle regulatory genes and the tumour immune microenvironment. The opposing correlations of CDK2 (negative) and CDK5 (positive) with regulatory *T* cells suggest different CDKs may regulate immune suppression through distinct mechanisms. The widespread negative correlations between multiple CDKs and naive *B* cells indicate potential roles in *B* cell development or function regulation. The diverse correlation patterns between different CDKs and NK cell and macrophage subsets point to connections between cell cycle regulation and innate immune cell function. Additionally, CD209's specific correlations with various immune cells suggest its potential role in modulating tumour immune responses. These complex association patterns provide crucial insights into immune regulatory mechanisms in non‐small cell lung cancer and may inspire new therapeutic strategies targeting both cell cycle and immune‐related pathways.

## 4 Discussion

Non‐small cell lung cancer (NSCLC) remains a global health challenge, characterised by complex molecular mechanisms and persistent high mortality rates despite therapeutic advances [20, 21]. Against this background, our study provides comprehensive insights into the inflammatory landscape and immune microenvironment of NSCLC, addressing critical knowledge gaps in understanding the disease pathogenesis.

The chronic inflammatory state has long been recognised as a critical contributor to carcinogenesis, creating a permissive environment for tumour initiation and progression through sustained production of inflammatory mediators, reactive oxygen species, and DNA damage. Our Mendelian randomisation analysis extends this understanding by establishing direct causal relationships between specific inflammatory factors and NSCLC risk [22, 23]. Particularly noteworthy is our identification of TGFB1 and CCL11 as risk‐promoting factors (OR = 1.173, *p* = 0.020; OR = 1.192, *p* = 0.003), which aligns with existing knowledge of their biological functions in cancer development. TGFB1's dual role—initially suppressing tumour development but later promoting invasion through immunosuppression and epithelial‐mesenchymal transition—illustrates the context‐dependent nature of inflammatory mediators in cancer progression. Similarly, CCL11's contribution to tumour progression through eosinophil recruitment and angiogenesis modulation represents an important mechanistic link in inflammation‐driven carcinogenesis.

Conversely, our findings regarding the protective effects of CD40 and CCL4 (OR = 0.857, *p* = 0.015; OR = 0.896, *p* = 0.049) highlight the complexity of immune‐inflammatory interactions in cancer. These molecules likely contribute to anti‐tumour immunity through distinct mechanisms—CD40 by enhancing dendritic cell activation and cytotoxic *T* cell responses, and CCL4 by potentially recruiting anti‐tumour immune cells to the tumour microenvironment. These findings reinforce the emerging paradigm that inflammatory responses can exert both tumour‐promoting and tumour‐suppressing effects, depending on the specific mediators involved and the stage of carcinogenesis.

The tumour microenvironment (TME) in NSCLC, as revealed by our analysis, demonstrates significant remodelling compared to normal lung tissue. The observed increases in CD8+ *T* cells alongside regulatory *T* cells (Tregs) represent a classic example of the competing forces within the TME—active anti‐tumour immunity counterbalanced by immunosuppressive mechanisms. This balance ultimately determines tumour progression trajectories and therapeutic responses. The decreased proportion of naive *B* cells in NSCLC samples further points to alterations in humoral immunity, an aspect of cancer immunology that has gained increased recognition in recent years [24, 25, 26].

Our network pharmacology approach, identifying 74 overlapping targets between drug associations and disease pathways, provides a systems‐level view of potential intervention points. The enrichment of inflammation‐related signalling cascades—IL‐17 pathway, TNF signalling, complement activation—further emphasises the centrality of inflammatory processes in NSCLC pathogenesis and offers potential targets for therapeutic development.

Perhaps most novel in our findings is the previously underappreciated connection between cell cycle control and immune regulation. The differential correlations between cyclin‐dependent kinases (CDKs) and immune cell populations—particularly the negative correlations of CDK2 and CDK3 with Tregs and the positive correlation between CDK5 and Tregs—suggest that cell cycle regulators may directly influence immunomodulatory functions. This observation aligns with emerging evidence that cancer cell‐intrinsic pathways can shape the tumour immune microenvironment and potentially influence responses to immunotherapy.

The most clinically significant discovery involves the previously unrecognised interplay between cell cycle machinery and immune surveillance mechanisms. The opposing relationships between CDK2/CDK3 (negative) and CDK5 (positive) with regulatory *T* cell populations suggest that selective CDK inhibition could serve dual therapeutic functions—blocking tumour proliferation while simultaneously enhancing anti‐tumour immunity. This finding provides a molecular rationale for combining CDK inhibitors with immune checkpoint blockade, potentially addressing the 60%–70% of NSCLC patients who fail to respond to immunotherapy alone.

The machine learning models achieving clinically meaningful discrimination (AUC 0.723–0.763) offer immediate utility for clinical decision‐making tools. These algorithms could be integrated into electronic health records to assist oncologists in risk assessment and treatment selection, particularly valuable in community practice settings where access to comprehensive molecular profiling may be limited. Further enhancement through incorporation of radiological features and routine laboratory parameters could yield robust clinical prediction platforms.

### 4.1 Limitations

However, several methodological limitations constrain immediate clinical translation. Our Mendelian randomisation instruments may inadequately capture the dynamic, context‐dependent nature of inflammatory factor activities across different disease stages and treatment contexts. The computational immune cell deconvolution approach, while informative, provides population‐level estimates that may not reflect individual patient heterogeneity—a critical consideration for personalised medicine applications. Additionally, our correlation‐based findings regarding CDK‐immune interactions lack experimental validation of underlying molecular mechanisms, limiting our understanding of whether these relationships represent direct causal pathways or indirect associations mediated by unknown factors.

## 5 Conclusion

This study addresses critical clinical challenges in NSCLC management by providing evidence‐based molecular insights that directly inform patient care strategies. Our findings establish clinically actionable pathways linking inflammation, immune dysfunction and cell cycle dysregulation in NSCLC pathogenesis.

## Author Contributions

**Yuan Fang:** data curation (equal), writing – original draft (equal). **Yuli Wang:** data curation (equal), writing – original draft (equal). **Lanlan Yang:** investigation (equal), validation (equal). **Si Yuan:** methodology (equal), writing – original draft (equal). **Jiefei Gu:** methodology (equal), writing – original draft (equal). **Ziyi Zhou:** investigation (equal), visualization (equal). **Zhihong Fang:** methodology (equal). **Yan Li:** formal analysis (equal), validation (equal), writing – review and editing (equal).

## Conflicts of Interest

The authors declare no conflicts of interest.

## Data Availability

All data used in this study are publicly available from TCGA, GEO (e.g., GSE278343), and the IEU OpenGWAS Project.
